# Resistance and resilience responses of a range of soil eukaryote and bacterial taxa to fungicide application

**DOI:** 10.1016/j.chemosphere.2014.03.031

**Published:** 2014-10

**Authors:** Christopher C. Howell, Sally Hilton, Kirk T. Semple, Gary D. Bending

**Affiliations:** a School of Life Sciences, University of Warwick, Gibbet Hill Road, Coventry, Warwickshire CV4 7AL, UK; b Lancaster Environment Centre, Lancaster University, Lancaster LA1 4YQ, UK

**Keywords:** Resistance, Resilience, Azoxystrobin, T-RFLP

## Abstract

The application of plant protection products has the potential to significantly affect soil microbial community structure and function. However, the extent to which soil microbial communities from different trophic levels exhibit resistance and resilience to such compounds remains poorly understood. The resistance and resilience responses of a range of microbial communities (bacteria, fungi, archaea, pseudomonads, and nematodes) to different concentrations of the strobilurin fungicide, azoxystrobin were studied. A significant concentration-dependent decrease, and subsequent recovery in soil dehydrogenase activity was recorded, but no significant impact on total microbial biomass was observed. Impacts on specific microbial communities were studied using small subunit (SSU) rRNA terminal restriction fragment length polymorphism (T-RFLP) profiling using soil DNA and RNA. The application of azoxystrobin significantly affected fungal and nematode community structure and diversity but had no impact on other communities. Community impacts were more pronounced in the RNA-derived T-RFLP profiles than in the DNA-derived profiles. qPCR confirmed that azoxystrobin application significantly reduced fungal, but not bacterial, SSU rRNA gene copy number. Azoxystrobin application reduced the prevalence of ascomycete fungi, but increased the relative abundance of zygomycetes. Azoxystrobin amendment also reduced the relative abundance of nematodes in the order Enoplia, but stimulated a large increase in the relative abundance of nematodes from the order Araeolaimida.

## Highlights

• We studied the resistance and resilience of soil microbial communities.
• There was a significant concentration-dependent impact on dehydrogenase activity.
• Significant impacts on nematode and fungal communities were also observed.

## 1 Introduction

Every community of living organisms is subjected to a range of stresses that can potentially deleteriously impact some or all of the species present, with the potential to affect community structure, function and/or diversity (Allison and Martiny, 2008). Such community responses can be considered in terms of resistance, which refers to the capacity of a community to maintain its size, composition, and function in the presence of a disturbance, and resilience, which describes the ability of an impacted community to recover its initial structure and function following a disturbance (Seybold et al., 1999; Allison and Martiny, 2008).

Giller et al. (1998) proposed two possible relationships between stress levels and microbial community diversity: an “extinction” relationship in which the diversity of a community is negatively correlated to an increase in stress levels, and a “competitive exclusion” relationship in which there is a hump-backed response to stress. In a hump-backed response, a mild stress would enhance the removal of dominant organisms, and promote an increase in diversity as other (normally less-abundant organisms) proliferate to fill the niche. However, there is limited experimental data to support these responses. It has been suggested that the resistance and/or resilience of soil communities to disturbances could be influenced by the initial biodiversity of a particular system. Girvan et al. (2005) observed greater resilience to benzene application in soil with a higher natural diversity, as demonstrated by a quicker recovery in the mineralisation rate of 2,4 dichlorophenol (2,4-DCP), than in lower diversity soil.

Some previous research has indicated the presence of “competitive exclusion” diversity responses to some stresses e.g. copper or cadmium amendment (Degens et al., 2001; Zhang et al., 2009b, 2010). However, whether such relationships apply following the addition of organic chemicals such as pesticides remains unknown.

The effects of pesticides on non-target organisms and the wider environment as a whole have been a concern for many years due to the biologically active nature of the compounds (Bending et al., 2007). Such non-target effects may result from either the direct toxicity of the compound, or as indirect impacts caused by the removal and/or increased proliferation of other species. It is thought that microbial communities may have lower natural resistance and/or resilience to pesticide impacts than plants and other larger organisms (Allison and Martiny, 2008).

Indeed, previous research using a range of broad-scale and molecular methods has shown that pesticides can significantly alter microbial community structure in different environments (Engelen et al., 1998; El Fantroussi et al., 1999; Wang et al., 2008; Zhang et al., 2009a). However, such studies have primarily been limited to bacterial and fungal communities. In particular, there is a paucity of information available about the impacts of pesticides on higher trophic level soil microorganisms such as nematodes and protozoa. Such organisms are integral members of soil food webs as both predators and prey and their activities are beneficial to nutrient cycling within the soil (Mikola et al., 2002), with the potential to impact plant growth (Bonkowski, 2004). Culture-dependent methods have previously shown impacts of pesticides on non-target eukaryotic microorganisms in soils (Ekelund, 1999; Ekelund et al., 2000; Boucard et al., 2004). However, such studies are limited by the fact that only a small percentage of soil microorganisms are culturable (Janssen et al., 2002).

There has been limited use of culture-independent molecular methods to investigate the non-target effects of pesticides on eukaryotic soil microorganisms. Bending et al. (2007) showed that three fungicides each had specific effects on eukaryote communities, apparently reducing the abundance of specific taxa. However, these effects occurred in the absence of impacts on broad-scale measurements such as microbial biomass. Similarly, Adetutu et al. (2008) found that the fungicide azoxystrobin altered the structure of soil fungal communities with impacts still observed up to 84 d after application, by which time over 60% of the applied compound had been degraded.

The current study investigated the impacts of pesticide application on the resistance and resilience responses of soil microbial communities from different trophic levels (bacteria, fungi, archaea, pseudomonads, and nematodes) using the strobilurin fungicide azoxystrobin as a model compound.

The strobilurin group of fungicides represent one of the most important groups of pesticides currently in use worldwide for the control of fungal crop pathogens. In 1999, sales of strobilurins totalled US$620 million worldwide (Bartlett et al., 2002) and this had increased to US$1.636 billion by 2007 (Stanley Alliance Info-Tech, 2011). Their structures are based on those of natural products secreted by wood-degrading basidiomycete fungi such as *Oudemansiella mucida* and *Strobilurus tenacellus* and can be either fungicidal or fungistatic. Azoxystrobin acts by binding to the ubiquinone (Qo) site of cytochrome b which forms part of the cytochrome bc_1_ complex in the fungal mitochondrial membrane. This binding disrupts the transfer of electrons from the cytochrome b portion of the complex, to the c_1_ portion, which stops the mitochondria producing ATP for the cell (Bartlett et al., 2002). Despite their widespread use, little is known about the effects of azoxystrobin and other strobilurin compounds on soil microbial communities, particularly with reference to non-target organisms.

Soil biomass-N and dehydrogenase activity analyses were performed to give an indication of broad-scale impacts, whilst molecular methods were used to determine the impacts of azoxystrobin concentration on the structure and diversity of specific microbial groups from different trophic levels. HPLC analysis was used to monitor azoxystrobin degradation/dissipation over the course of the experiment.

## 2 Materials and methods

### 2.1 Soil collection and preparation

Soil was collected from Hunts Mill field at the Wellesbourne Campus of the University of Warwick School of Life Sciences, UK, during January 2008. The soil is a sandy loam of the Wick series with a composition of 73.4% sand, 12.3% silt, and 14.3% clay (Bending et al., 2007). The field had been managed as set-aside for over a decade and thus had received no pesticide applications. Soil was collected from the top 20 cm to comply with OECD guidelines for soil sampling in agricultural soils (OECD, 2011). Prior to azoxystrobin application, the soil was re-wetted to a matric potential of −33 kPa (Bending et al., 2007). This equated to a soil moisture content of 13.5%.

### 2.2 Azoxystrobin addition to soil

Azoxystrobin (Greyhound Chromatography, Birkenhead, UK) was dissolved in acetone and added to the soil at a solvent:soil ratio of 1:20 (Northcott and Jones, 2000), giving concentrations of 1, 5, 10 and 25 mg kg^−1^ soil, with 5 mg kg^−1^ representing the UK maximum recommended dose of azoxystrobin in the top cm of soil (Bending et al., 2007) and therefore the maximum dose which could reach the soil either directly, such as from spraying prior to canopy closure, or indirectly, following residue wash-off from the canopy. A total of 2.4 kg of soil was required for each pesticide concentration. The azoxystrobin solution was initially applied to one quarter of the soil and mixed with a sterile stainless steel spoon. The soil was then stored at room temperature in a fume hood for 2 h to allow evaporation of the acetone. The remaining three quarters were then mixed in gradually over a 10 min period to ensure an even distribution of the compound throughout the soil (Doick et al., 2003). Control soils were amended in the same way as the treated soils, but without azoxystrobin. 120 g Portions were then transferred to sterile 250 mL glass Duran bottles, wrapped in aluminium foil and stored at 15 °C in the dark. 4 Replicates of each treatment were destructively sampled at time 0, and then on a monthly basis for 4 months.

### 2.3 Effects on broad-scale microbial properties

Soil biomass-N was measured using the CHCl_3_ fumigation method of Joergensen and Brookes (1990). Obtained ninhydrin-N values were converted to biomass-N using a conversion factor of 3.1 (Amato and Ladd, 1988). Dehydrogenase activity was monitored as detailed by Tabatabai (1994).

### 2.4 Azoxystrobin extraction and analysis

10 g of azoxystrobin-amended soil was added to 50 mL centrifuge tubes and mixed with 20 mL of HPLC-grade acetonitrile (Fisher Scientific, UK). The tubes were shaken by hand and placed on a shaker for 1 h. Following shaking, the samples were left for 30 min to settle and then centrifuged at 4000 rpm for 2 min. 2 mL Of the supernatant was transferred into a 2 mL screw-top glass HPLC vial (Chromacol Ltd., UK). Samples were analysed using an Agilent 1100 series unit with a diode array detector (DAD) and LiChrospher® 100 RP-18e (5 μm) HPLC column (Column length: 125 mm, Pore size: 100 Å, Internal diameter: 4.00 mm) (Agilent, UK). A liquid phase composed of 75% HPLC-grade acetonitrile (Fisher Scientific, UK) and 25% distilled water was used at a flow rate of 1.30 mL min^−1^. 25 μL of each sample was injected and the concentration of azoxystrobin determined by monitoring the absorbance at 230 nm. The limit of detection (LOD) of the equipment was 0.04 μg g^−1^ soil and the limit of quantification (LOQ) was 0.1 μg g^−1^ soil.

### 2.5 Culture-independent analyses of microbial community structure changes

DNA was extracted from the soil samples using a FastDNA® Spin Kit (Qbiogene, UK). PCR reactions were set up containing MegaMix ready PCR Mix (Microzone Ltd., UK) using the manufacturer’s guidelines, and a fluorescently-labelled primer pair specific to the microbial community being studied (Table S1). Total RNA was extracted from 0 and 25 mg kg^−1^ soils 1-month post-amendment using the FastRNA® Pro Soil-Direct Kit (Qbiogene, UK) and reverse transcriptase PCR (RT-PCR) was performed using the Qiagen® OneStep RT-PCR Kit (Qiagen, UK). The primer pairs used for RT-PCR were: EF4f-FAM/EF3r, 63f-NED/1087r-VIC, and Nem18Sf-VIC/Nem18Sr (Table S1). All labelled primers were obtained from Applied Biosystems, Warrington, UK and all un-labelled primers from Invitrogen, Paisley, UK. PCR products were prepared for T-RFLP analysis as described by Hunter et al. (2010). T-RFLP analysis was carried out using an Applied Biosystems 3130XL Genetic Analyzer (Applied Biosystems, Warrington, UK), and the results analysed using GeneMarker® software (Softgenetics®, USA). TRF sizes were determined by reference to LIZ-1200 standards, and the default software settings. Only peaks with intensity values of 50 or over were used for further analysis (Hackl et al., 2004).

### 2.6 Clone library production and sequence analysis

Clone libraries were produced for nematodes and fungi from 0 mg kg^−1^ and 25 mg kg^−1^ treatment RNA extracts at the 1 month time point as described by Hunter et al. (2010). Forward and reverse sequences from each clone were determined using the primers M13R and M13F (O’Shaughnessy et al., 2003). Sequencing was performed using an Applied Biosystems 3130XL Genetic Analyzer (Applied Biosystems, UK). Sequences were analysed using the SeqMan™ programme (DNASTAR Inc., USA). The sequences were aligned and the insert sequence determined. Sequence homologies were identified using the nucleotide BLAST (NCBI, url: http://blast.ncbi.nlm.nih.gov/) database. 80 and 83 18S rRNA sequences were obtained from the 0 and 25 mg kg^−1^ fungal samples, respectively. For nematodes, 81 and 79 sequences were obtained from the 0 and 25 mg kg^−1^ treatments, respectively. The EditSeq™ programme (DNASTAR Inc.) was used to determine the position of *Hha*I and *Msp*I (fungi), and *Aci*I and *Hae*III (nematodes) restriction sites in each sequence.

### 2.7 Quantitative PCR analysis of bacterial and fungal DNA samples

Quantitative PCR (qPCR) analysis was carried out for 0 mg kg^−1^ and 25 mg kg^−1^ bacterial (16S rRNA) and fungal (18S rRNA) DNA samples, 1 month post application. qPCR reactions were set up using the 2× SYBR Green mix (Applied Biosystems, UK) according to the manufacturer’s instructions. Each qPCR reaction was carried out in triplicate. The primer pairs Eub338f/Eub518r (Fierer et al., 2005) and 5.8s/ITS1F (Gardes and Bruns, 1993) were used for bacterial and fungal community analysis, respectively. All primers were obtained from Invitrogen™. Fungal and bacterial standard samples produced from existing clone libraries were first sequenced as described in Section 2.6. The bacterial standard had a 97% sequence homology to the uncultured α-Proteobecterium clone AKYH1384 (AY921937.1), and the fungal standard had a 98% sequence homology to the species *Phoma exigua* var. exigua (AB454232.1). Purified products were obtained using the QIAfilter Plasmid Midi Kit (Qiagen, UK) using the manufacturer’s instructions. qPCR reactions were carried out using an ABI Prism 7900 HT sequence detection system (Applied Biosystems, UK). All samples were analysed using the SDS 2.1 software (Applied Biosystems). qPCR samples were considered successful if the *R*^2^ value was >0.99 and the slope value between −3.0 and −3.4. qPCR products were run on a 1% agarose gel to ensure that only the intended fragment was amplified. Mean quantities (in pg μL^−1^), and standard deviations were calculated for each triplicate set using Microsoft Excel 2007 (Microsoft, USA). DNA copy numbers were calculated as described by Whelan et al. (2003).

### 2.8 Statistical analyses

Two-way analysis of variance (ANOVA) with contrast analysis was used to determine significant differences between treatments over the experimental period. The factors used in the ANOVA were azoxystrobin concentration and sampling time. An additional contrast function was added to the ANOVA using the GenStat Version 12 statistics programme (VSN International, UK) to enable the impacts of individual azoxystrobin concentrations to be compared to each other. An example of the GenStat code used for this analysis can be seen in Fig. S1.

The time taken for 50% of the applied azoxystrobin to degrade (DT_50_) was calculated using the vinterpolate function in GenStat Version 12 (VSN International, UK).

T-RFLP data was analysed using non-metric multi-dimensional scaling (NMDS), using Primer6 software (Primer-E Ltd., UK), in order to determine the impacts of pesticide concentration on microbial community structure. This method compares the dissimilarity between each sample and plots the distances between each sample on a 2D ordination plot (Ramette, 2007). Similarity percentage (SIMPER) analysis was used to determine which TRFs contributed to the community variation between defined treatments.

The significance of differences in community structure between 0 mg kg^−1^ and 25 mg kg^−1^ treatment bacterial and fungal clone libraries were determined using the Mann–Whitney *U*-Test page of the *Caenorhabditis elegans* WWW Server (url: http://elegans.som.vcu.edu/~leon/stats/utest.html).

2-way ANOVA and LSD analyses were used to identify significant differences in fungal and nematode SSU rRNA sequence copy numbers between treatments.

## 3 Results

### 3.1 Azoxystrobin recovery using HPLC

**Fig. 1:**
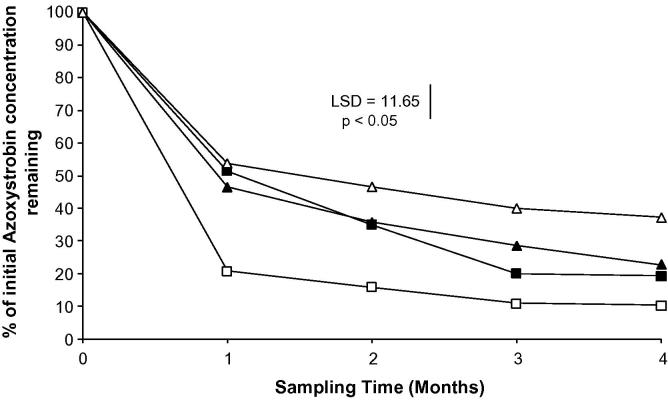
Recovery of azoxystrobin. ■ 1 mg kg^−1^ □ 5 mg kg^−1^ ▴ 10 mg kg^−1^ Δ 25 mg kg^−1^. Each data point represents the mean of 4 experimental replicates.

In all azoxystrobin treatments, degradation proceeded rapidly within the first month post-application, but had almost ceased after 3 months. The degradation rate in the 25 mg kg^−1^ treatment was slower than the other treatments, whereas the compound degraded most quickly in the 5 mg kg^−1^ treatment. DT_50_ values ranged from 19 (5 mg kg^−1^) to 47 (25 mg kg^−1^) d. After 4 months, between 10% and 37% of the applied azoxystrobin remained across the different treatments (Fig. 1).

### 3.2 Analysis of broad-scale microbial properties

Azoxystrobin concentration had no significant effect on biomass-N over the course of the experiment (*p* = 0.149). Average biomass-N values during the 4-month experimental period ranged from 112.8 to 228.8 μg g^−1^ soil (Data not shown).

**Fig. 2:**
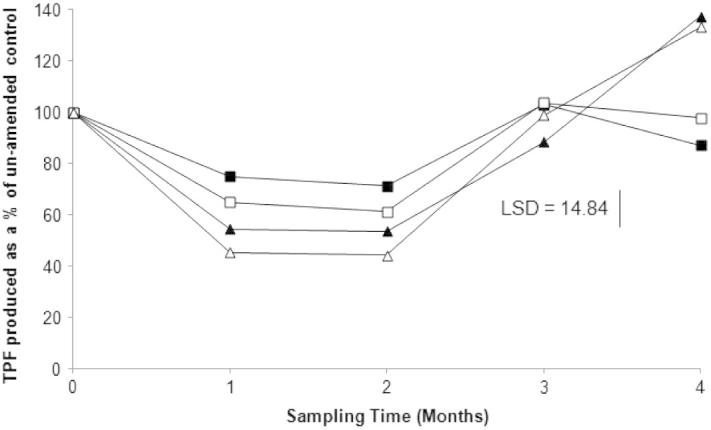
Percentage dehydrogenase activity in azoxystrobin-amended soils compared to 0 mg kg^−1^ controls. TPF = triphenyl formazan. ■ 1 mg kg^−1^ □ 5 mg kg^−1^ ▴ 10 mg kg^−1^ Δ 25 mg kg^−1^. Each data point represents the mean of 4 experimental replicates.

Soil dehydrogenase activity was significantly affected by the concentration of azoxystrobin applied (*p* < 0.01) (Fig. 2). There was a marked decrease in dehydrogenase activity in all treatments after 1 month, relative to the control. At this time point dehydrogenase activity ranged from 75% of the un-amended control in the 1 mg kg^−1^ treatment, to only 45% in the 25 mg kg^−1^ samples. Dehydrogenase activity remained at these levels up to the 2 month sampling point. However, by 3 months, there had been a marked increase in dehydrogenase activity in all treatments. Indeed, the 1 and 5 mg kg^−1^ treatments recorded activity levels that were 102.9% and 103.6% those of the control. Values of 88.4% and 98.8% were recorded for 10 and 25 mg kg^−1^ treatments respectively. After 4 months, dehydrogenase activity in the 10 and 25 mg kg^−1^ treatments were higher than those of the controls (137.4% and 133.2%, respectively). The 1 and 5 mg kg^−1^ treatments recorded values 87.1% and 97.8% that of the control.

### 3.3 T-RFLP analysis of microbial community structure

#### 3.3.1 Fungi

**Fig. 3:**
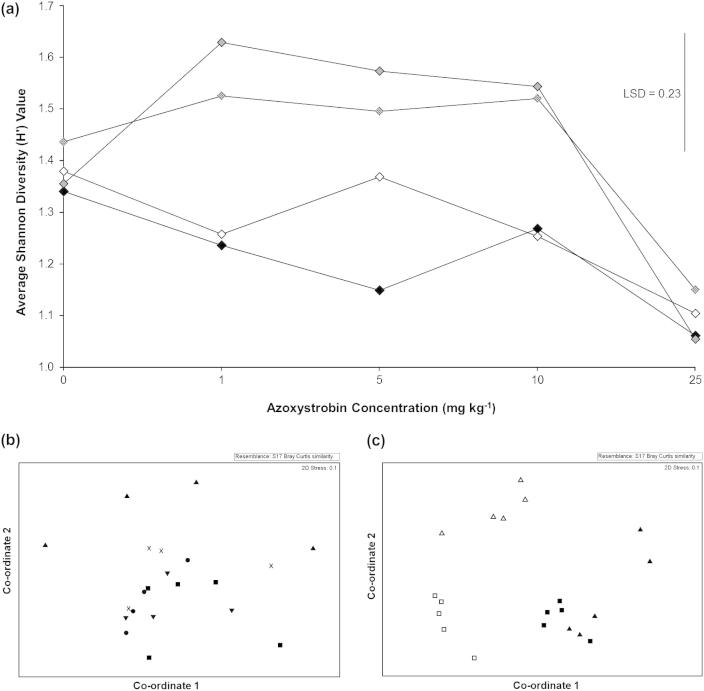
(a) The average Shannon diversity index (H′) of fungal communities recorded using the EF4f/EF3r primer pair over 4 months following azoxystrobin application. ♦ 1 month ♢ 2 months  3 months  4 months. Each data point represents the mean of 4 experimental samples. (b) NMDS analysis of fungal community structure determined using the primer pair EF4f and EF3r and grouped by Azoxystrobin concentration. ■ 0 mg kg^−1^ ▾ 1 mg kg^−1^ ● 5 mg kg^−1^ × 10 mg kg^−1^ ▴25 mg kg^−1^. Each data point represents the mean of 4 experimental replicates at an individual time point. (c) NMDS analysis of fungal community structures using extracted DNA and RNA 1 month post azoxystrobin application. Each data point represents an experimental replicate. ■ 0 mg kg^−1^ DNA □ 25 mg kg^−1^ DNA ▴ 0 mg kg^−1^ RNA Δ 25 mg kg^−1^ RNA.

There was no significant difference in the overall number of TRFs recorded in the different treatments (*p* = 0.430) or across the different sampling times (*p* = 0.080). 64 unique TRFs were observed, with between 12 and 24 recorded in an individual trace (data not shown). The diversity in the 25 mg kg^−1^ treatment was significantly lower than all of the other treatments (*p* < 0.01), recording values between 78% and 80% those of the unamended controls for each time point (Fig. 3a). No significant differences in diversity were observed between any of the other treatments.

Although ANOSIM analysis showed no overall significant impact of azoxystrobin on fungal community structure (*p* = 0.168), pair-wise analysis did show that the application of azoxystrobin at a concentration of 25 mg kg^−1^ significantly changed the fungal community structure, relative to the control (*p* = 0.032), 1 (*p* = 0.029), and 5 mg kg^−1^ (*p* = 0.029) treatments across the experimental period (Fig. 3b). Sampling time did not significantly affect the fungal community structure (*p* = 0.738). TRFs at 143, 146, and 148 bp (*Hha*I) were dominant in the 0–10 mg kg^−1^ concentrations, but were absent in the 25 mg kg^−1^ treatment. SIMPER analysis identified that the absence of these TRFs was responsible for approximately 12% (143 bp), 23% (146 bp), and 17% (148 bp) of the total community structure variation between the 25 mg kg^−1^ and the 0–10 mg kg^−1^ treatments.

T-RFLP analysis of RNA extracts taken from 0 and 25 mg kg^−1^ treatments 1 month post-application was used to determine if azoxystrobin significantly affected the active fungal community. The application of azoxystrobin significantly affected the numbers of TRFs (*p* = 0.04) (Table S2) with amended samples having, on average, 53% the number of TRFs of the unamended controls. This compares with 67% recorded for the DNA-derived samples. Furthermore, NMDS (Fig. 3c) and ANOSIM analyses showed that azoxystrobin significantly affected the active fungal community structure (*p* < 0.01) whilst fungal diversity was also significantly affected (Table 1). The NMDS plot showed no significant difference in the control and 25 mg kg^−1^ treatments for the DNA-derived samples. However, there was a significant difference in fungal community structure between the control and 25 mg kg^−1^ treatments for the RNA-derived samples. Furthermore, the DNA- and RNA-derived samples also formed clearly distinct groups. This suggested significant differences in the structures of these groups. ANOSIM analyses supported the NMDS plot observations. There was a significant difference in community structure between amended and un-amended samples for both DNA- (*p* = 0.046) and RNA-derived samples (*p* = 0.024). There was also a highly significant difference in the community structure of samples produced using extracted DNA or RNA (*p* < 0.01).

#### 3.3.2 Nematodes

**Fig. 4:**
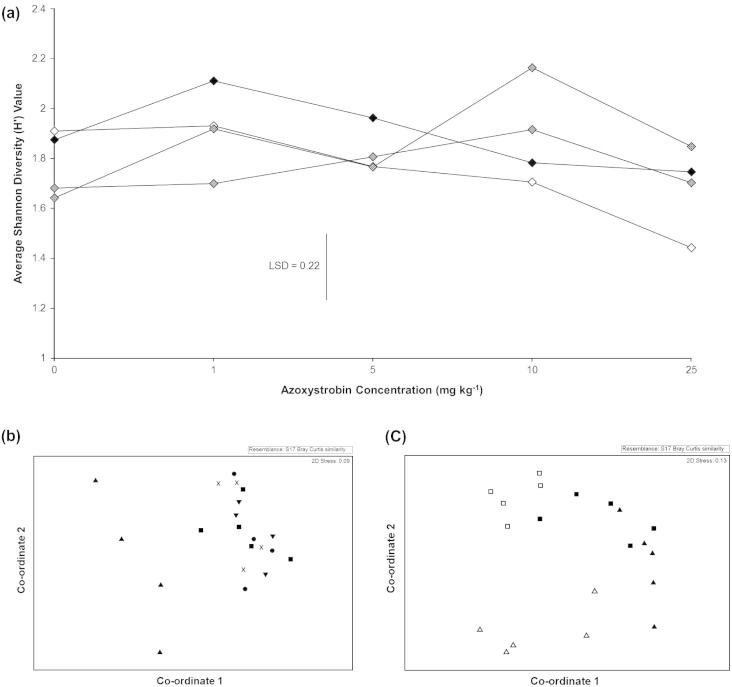
(a) The average Shannon diversity index (H′) of nematode communities recorded using the Nem 18Sf/Nem 18Sr primer pair over 4 months following azoxystrobin application. ♦ 1 month ♢ 2 months  3 months  4 months. Each data point represents the mean of 4 experimental samples at an individual time point. (b) NMDS analysis of fungal community structure determined using primer pair EF4f and EF3r and grouped by Azoxystrobin concentration. ■ 0 mg kg^−1^ ▾ 1 mg kg^−1^ ● 5 mg kg^−1^ × 10 mg kg^−1^ ▴ 25 mg kg^−1^. Each data point represents the mean of 4 experimental replicates. (c) NMDS analysis of fungal community structures using extracted DNA and RNA 1 month post azoxystrobin application. Each data point represents an experimental replicate. ■ 0 mg kg^−1^ DNA □ 25 mg kg^−1^ DNA ▴ 0 mg kg^−1^ RNA Δ 25 mg kg^−1^ RNA.

A total of 67 different nematode TRFs were obtained with between 8 and 29 being recorded in a single trace. There was no significant impact of azoxystrobin concentration (*p* = 0.538) or sampling time (*p* = 0.537) on the number of nematode TRFs. However, azoxystrobin concentration did significantly impact nematode community diversity (*p* = 0.049) (Fig. 4a). Contrast analysis showed that this effect was due to highly significant differences in diversity in the 25 mg kg^−1^ treatment compared to the other treatments (*p* < 0.01).

ANOSIM analysis showed that azoxystrobin application significantly affected the nematode community structure (*p* = 0.019). Pair-wise comparisons showed that this was due to the community structure in the 25 mg kg^−1^ treatment being significantly different to those of the control (*p* < 0.01), 1 mg kg^−1^ (*p* = 0.02), 5 mg kg^−1^ (*p* = 0.03), and 10 mg kg^−1^ (*p* = 0.03) treatments (Fig. 4b). There was no significant effect of sampling time on nematode community structure (*p* = 0.615).

Two of the recorded TRFs, 421 bp (*Aci*I) and 458 bp (*Hae*III), showed dramatic reductions in intensity in the 25 mg kg^−1^ samples, compared to the other treatments. Indeed, TRF 421 bp which was present in all of the 0–10 mg kg^−1^ treatments, was absent from all of the 25 mg kg^−1^ treatments, whilst TRF 458 bp was present in all of the 0–10 mg kg^−1^ treatments, but was only present in the 25 mg kg^−1^ treatment at the 1 month time point. SIMPER analysis showed that the absence of these two TRFs was responsible for an average of 13% (421 bp) and 12% (458 bp) of the total community structure variation between the 25 mg kg^−1^ and 0–10 mg kg^−1^ treatments.

As with the fungal samples, azoxystrobin had a significant impact on RNA-derived nematode TRF numbers (*p* = 0.018) (Table S2). RNA from the amended samples contained an average of 70% the TRF numbers present in the un-amended controls. However, there was no significant difference in the RNA-derived active nematode diversity between the control and 25 mg kg^−1^ treatments (*p* = 0.249) (Table 1). NMDS and ANOSIM analyses showed that there was a significant difference in community structure when the DNA- and RNA-derived samples were compared (*p* < 0.01). Furthermore, azoxystrobin application had a significant effect on the structure of the active nematode community in the RNA-derived analysis (Fig. 4c) (*p* < 0.01). However, no significant differences between nematode community structure in DNA-derived samples were observed (*p* = 0.211).

#### 3.3.3 Bacteria

A total of 159 individual TRFs were recorded for the bacterial samples. In a single trace, the number of TRFs varied markedly from 33 to 109. Neither azoxystrobin concentration (*p* = 0.994) nor sampling time (*p* = 0.123) had a significant impact on bacterial TRF numbers. There was also no significant impact of azoxystrobin concentration on bacterial community structure (*p* = 0.636) or diversity (*p* = 0.292). RNA-derived analysis also showed no significant impacts of azoxystrobin on active bacterial diversity (Table 1), TRF numbers, or community structure (data not shown).

#### 3.3.4 Archaea

T-RFLP analysis produced a total of 98 individual archaeal TRFs with between 8 and 33 being present in an individual trace. Azoxystrobin concentration was found to have no significant impacts on archaeal TRF numbers (*p* = 0.352), diversity (*p* = 0.754), or community structure (*p* = 0.890) (Data not shown).

#### 3.3.5 Pseudomonads

A total of 73 individual pseudomonad TRFs were recorded with between 9 and 19 being present in an individual trace. However, azoxystrobin concentration did not have a significant impact on the pseudomonad community with *p* values of 0.905, 0.147, and 0.941 recorded for effects on TRF numbers, community diversity, and community structure, respectively (data not shown).

### 3.4 Fungal and nematode clone library analysis

In order to gain a greater insight into the impacts of azoxystrobin on soil fungal and nematode populations, 18S rRNA gene clone libraries were produced from the 1 month RNA samples from the 25 mg kg^−1^ treatment, and the un-amended controls.

**Fig. 5:**
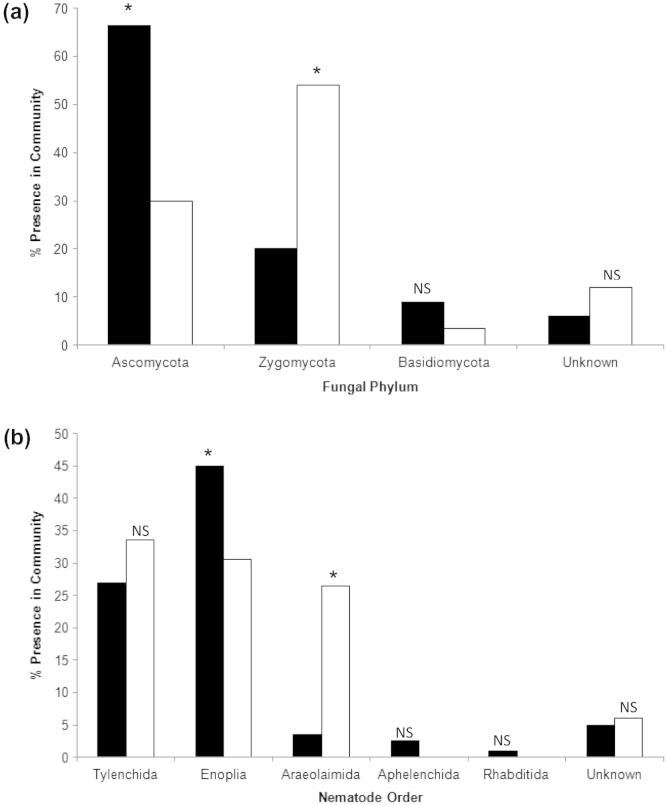
The prevalence of different (a) fungal and (b) nematode groups within clone libraries produced from 0 and 25 mg kg^−1^ (1 month) samples. ■ 0 mg kg^−1^ □ 25 mg kg^−1^. NS = no significant difference between the treatments. * = Significant difference between treatments (*p* < 0.05).

Analysis of the fungal clone libraries using the Mann Whitney *U*-Test indicated that azoxystrobin application had a significant impact on community structure (*p* = 0.017). The fungal libraries consisted of 80 individual sequences for the 0 mg kg^−1^ treatment and 83 for the 25 mg kg^−1^ samples. The 0 mg kg^−1^ library was composed of 66.5% ascomycetes, 20% zygomycetes, 9% basidiomycetes, and 6% that showed sequence homology to “uncultured fungi” (Fig. 5a). In contrast, in the 25 mg kg^−1^ library only 30% of the sequences showed homology to ascomycetes whilst there was an increase in the percentage of zygomycetes to 54%. This change was mostly due to an increase in the prevalence of sequences showing a homology to *Zygomycete* sp. AM-2008a (EU428773.1; EU428769.1), from 9.0% in the un-amended treatment library to 49.5% in the 25 mg kg^−1^ library. Basidiomycete and “uncultured fungi” sequences accounted for 3.5% and 12% of the whole library, respectively.

Mann Whitney *U*-Test analysis of the nematode clone library data showed that azoxystrobin application had a significant effect on community structure (*p* = 0.026). The clone libraries included 81 and 79 sequences for the 0 mg kg^−1^ and 25 mg kg^−1^ treatments, respectively.

Taxonomic analysis of the 0 mg kg^−1^ clone library showed that the majority of the clones came from the orders Enoplida (45%) and Tylenchida (27%). The remainder were from the orders Araeolaimida (3.5%), Aphelenchida (2.5%), and Rhabditida (1%). 5% of the clones were classified as “uncultured nematodes” (Fig. 5b). The most common sequence recorded showed homology to *Pratylenchus neglectus* (EU669924.1) which accounted for 26% of the clone library sequences. Sequences showing homologies to *Xiphinema rivesi* (HM923144.1), *Achromadora* sp. (AY593940.1) and *Trichistoma* sp. (GQ503079.1) constituted 17%, 13.5%, and 10% of the clone library sequences, respectively. Following azoxystrobin application the major change that occurred was an increase in prevalence of nematodes from the order Araeolaimida to 26.5% of the total clones. This was due to a large increase in the number of sequences showing homologies to the genus *Plectus* sp. (U617661.1) There was also an increase of 6.5% in the number of clones that showed sequence homologies to the order Tylenchida. Conversely, those with homologies to the order Enoplia decreased by 14.5%. In contrast to the control, there were no clones present that showed sequence homologies to the orders Aphelenchida or Rhabditida. The 25 mg kg^−1^ clone library consisted of 12 named genera. The dominant sequences in this library were related to *P.* *neglectus* (EU669924.1) (32.5% of the sequences), *Xiphinema* sp. (HM921342.1) (21% of the sequences), and *Plectus rhizophilus* (AY593929.1) (12.5% of the sequences). Sequences for the genera *Achromadora* were not found at all in the 25 mg kg^−1^ library and *Trichistoma* sp. sequences only represented 2.5% of the sequences (as opposed to 10% in the 0 mg kg^−1^ library). Conversely, the prevalence of *P.* *rhizophilus* increased by 9% in the 25 mg kg^−1^ library samples.

### 3.5 Quantitative polymerase chain reaction analysis

qPCR analysis was performed to determine the effects of azoxystrobin concentration on 16S (bacteria) and 18S (fungi) rRNA gene copy number. Samples were analysed 1 month post-application.

Azoxystrobin application did not have a significant effect on bacterial copy number (*p* = 0.622) (Standard curve slope: −3.102; *R*^2^ value: 0.991). An average bacterial copy number of 3.15 × 10^6^ was recorded for the 0 mg kg^−1^ control. Copy numbers in the amended samples ranged from 2.275 to 3.775 × 10^6^ (data not shown).

**Fig. 6:**
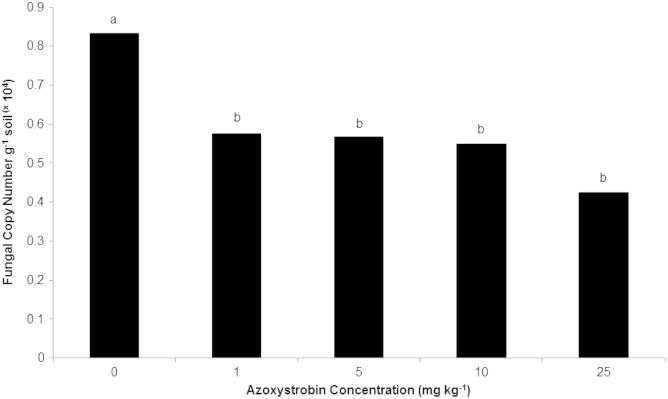
Average fungal copy numbers recorded for soils exposed to different Azoxystrobin concentrations 1 month prior to sampling. LSD = 0.223. Each copy number value represents the mean of 4 experimental replicates. Different letters denote significant differences between treatments.

In contrast, azoxystrobin application did have a significant impact on fungal copy number (*p* = 0.043) (Standard curve slope: −3.37; *R*^2^ value: 0.991). Fungal copy numbers in the un-amended controls averaged 0.83 × 10^4^ g^−1^ soil (Fig. 6). No significant differences in fungal copy number were observed between the 1, 5, 10 and 25 mg kg^−1^ treatments. Fungal copy numbers for the 1, 5, 10, and 25 mg kg^−1^ samples were 0.58, 0.57, 0.55, and 0.43 × 10^4^ g^−1^ respectively.

## 4 Discussion

In this study the different broad and fine scale methods gave sometimes contradictory indications of azoxystrobin impacts on soil microbial communities. 18S rRNA analysis of fungal and nematode communities produced largely complementary results indicating significant impacts only in the 25 mg kg^−1^ treatments across the 4 month experimental period. The exception to this was the fungal diversity analysis which showed a concentration dependent reduction in diversity, followed by a recovery in the 1–10 mg kg^−1^ treatments after 3 months. This result more closely matched that of the dehydrogenase analysis which showed a significant concentration dependent impact on activity after 1 and 2 months, followed by a recovery to the control levels by 3 months in the 1 and 5 mg kg^−1^ treatments. In contrast, no significant impacts on soil biomass-N were observed for any azoxystrobin concentration, at any sampling time. There were no apparent relationships between dehydrogenase activity, soil microbial biomass-N, microbial diversity, and azoxystrobin degradation.

NMDS analysis based on fungal and nematode 18S rRNA T-RFLP data and Shannon diversity analysis of the nematode community, showed a “threshold” response with no significant impacts observed in the 1–10 mg kg^−1^ treatments, but significant changes in community structure and reductions in diversity recorded in the 25 mg kg^−1^ treatments. A “threshold” response was also observed for the fungal qPCR analysis, but at a much lower concentration than was suggested by the diversity analysis, with all treatments exhibiting significantly lower SSU rRNA gene abundance than the unamended controls. These “threshold” relationships between stress levels and community diversity differs from both the “extinction” and “competitive exclusion” responses proposed by Giller et al. (1998). However, fungal community diversity only exhibited a “threshold” relationship after 1 and 2 months, after which a response similar to the “competitive exclusion” relationship proposed by Giller et al. (1998) was observed with the 1, 5, and 10 mg kg^−1^ treatments exhibiting significantly higher diversities than the control samples. Interestingly, this increase in fungal diversity after 3 months appears to correlate well with the observed recovery in soil dehydrogenase activity. In contrast, the diversity in the 25 mg kg^−1^ treatments remained significantly lower than the control at all of the sampling times.

Soil microbial biomass-N was not significantly affected by azoxystrobin application. NMDS and Shannon diversity analysis showed that the soil bacterial community was similarly unaffected, unlike the fungal and nematode communities. This may suggest that the chloroform fumigation assay used for biomass-N analysis preferentially represented a measure of the bacterial community. If so, this would differ from previous suggestions that fungal populations are more susceptible to chloroform fumigation than bacterial populations (Toyota et al., 1996). However, the effectiveness of biomass-N analysis as a measure of total biomass can be limited by potentially large variations in the N contents of different microbial groups (e.g. bacteria and fungi). As a result, future work may benefit from the use of biomass-C as a more reliable indicator of total biomass (Spedding et al., 2004).

In contrast, dehydrogenase activity showed a proportional, time-dependent effect, with increasing pesticide concentrations resulting in greater effects on the microbial community. This suggests that dehydrogenase analysis could have been a stronger indicator of impacts on nematode and/or fungal communities. In the 1 and 5 mg kg^−1^ treatments there was an initial decrease in dehydrogenase activity, which indicated that the microbial community had a low initial resistance to azoxystrobin application. However, by the 3 month sampling point dehydrogenase activity had returned to levels comparable to those observed in the un-amended controls. This suggests that the microbial community had recovered from the initial impact caused by the pesticide, despite the HPLC data showing that between 15% and 25% of the initially applied azoxystrobin still remained in the system, suggesting that there was not a direct link between dehydrogenase activity and azoxystrobin degradation. In contrast, in the 10 and 25 mg kg^−1^ treatments dehydrogenase activity was significantly higher than in the controls after 4 months. This increase in dehydrogenase activity is again indicative of community recovery following removal of the fungicide stress, but the fact that it was elevated over the control could also reflect either the removal of competitive interactions, or new microbial growth following the utilization of biomass killed by the azoxystrobin.

Both fungal and nematode communities were found to be susceptible to the application of azoxystrobin, particularly the 25 mg kg^−1^ treatment. Fungal community structure, diversity, and 18S rRNA gene copy number were all significantly impacted. Additionally, significant community structure and diversity impacts were also observed for nematode communities.

SIMPER analysis of fungal T-RFLP traces showed that the TRFs at 143, 146 and 148 bp predominated in the 0–10 mg kg^−1^ treatments but were absent in the 25 mg kg^−1^ samples. Predicted TRF sizes within this range were also present in the clone library produced from 0 mg kg^−1^ samples, but not in the 25 mg kg^−1^ treatment. These clones showed homologies to *Byssoascus striatosporus* (97%) (AB015776.1) along with the plant pathogens *Gibberella fujikuroi* (100%) (AB237662.1) and *Fusarium oxysporum* (99–100%) (JQ926985.1). All of these species are ascomycete fungi. This raises the possibility that ascomycete fungi may be more susceptible to azoxystrobin application. Indeed, of the 24 taxa from the clone library that decreased following the application of azoxystrobin, 22 were ascomycetes with the other two being basidiomycetes. This appears to contradict the widely-accepted view of the broad-spectrum nature of this compound. In contrast, there was a significant increase in the number of sequences showing homology to zygomycete fungi following azoxystrobin application. In particular, 49.5% of the sequences from the amended samples showed homology to *Zygomycete* sp. AM-2008a, compared to 9% in the un-amended samples. Many zygomycete species are fast-growing r-strategy fungi and this could explain their rapid growth to fill the niche left by the negatively impacted ascomycete fungi. In contrast, there were no significant changes in basidiomycete communities between the two treatments.

However, these changes in fungal community structure, diversity, and 18S rRNA gene copy number were not mirrored by significant changes in the overall bacterial, archaeal or Pseudomonad communities. This suggests that the bacterial community were neither competing with the fungi, nor using fungal biomass killed by the fungicide as a nutrient source.

Soil nematode communities were impacted by azoxystrobin application with both community structure and diversity found to be significantly affected. This is important as it represents a non-target impact of azoxystrobin on higher trophic level organisms. Sequences with homology to *X.* *rivesi* (HM921344.1), *Xiphinema chambersi* (HM191718.1), and *Bitylenchus dubius* (AY284601.1) decreased in abundance following the application of azoxystrobin, whereas sequences with homology to *P.* *neglectus* (EU669924.1) and an uncultured *Xiphinema* sp. (HM921342.1) became more prevalent. Unfortunately, the grazing habits and other traits of many of the nematode species identified by clone libraries in this paper remain unknown so it is not clear whether these changes reflect direct impacts of the fungicide, or indirect effects associated with changes to the biomass of fungal taxa on which some nematodes graze. This serves to emphasise that current knowledge of the fine-scale aspects of soil nematode community dynamics (especially in relation to responses to perturbation) is very limited, particularly in comparison with other microbial groups such as bacteria and fungi. Edel-Hermann and colleagues (2008) raised this point, and allied it to the fact that different microbial groups do not exist in isolation within the soil, but interact extensively in areas such as nutrient cycling, competition and predation. This represents an extensive knowledge gap considering the role that nematodes are considered to play in regulating the structure and function of the soil microbial community as a whole (Ekelund, 1999; Ekelund et al., 2000; Boucard et al., 2004).

## 5 Conclusions

The main aim of this work was to ascertain the impacts of pesticides on soil microbial resistance and resilience responses at different trophic levels using the widely-used, broad-spectrum fungicide azoxystrobin as a model compound. Azoxystrobin application significantly affected the structure and diversity of fungal and nematode communities over the 4-month period. The only evidence found to support either of the relationships between stress levels and community diversity proposed by Giller et al. (1998) was observed in the fungal diversity analysis after 3 months. Instead, the molecular analyses mostly appeared to show a “threshold” relationship where community diversity was unaffected between 0 and 10 mg kg^−1^. However, between 10 and 25 mg kg^−1^ the diversity decreased rapidly, with no apparent recovery. Similarly, there was a significant concentration-dependent impact on dehydrogenase activity. Resilience responses in dehydrogenase activity were recorded for the 1 and 5 mg kg^−1^, but not the 10 and 25 mg kg^−1^ treatments. However, bacterial, archaeal, and pseudomonad communities were unaffected by azoxystrobin application.

Current knowledge on the effects of pesticide application on non-target microbial communities in soils remains limited, particularly in relation to higher trophic level microorganisms. The work presented here gives an indication of the resistance and resilience of such communities following perturbation and how stress levels may affect community diversity. However, further research in this area could benefit from the use of meta-genomic approaches to study changes in microbial community structure (Urich et al., 2008) in response to strobilurin fungicide application, or changes in the expression of genes thought to be involved in stress responses.

## Figures and Tables

**Table 1:** Fungal, bacterial, and nematode Shannon diversity index (H′) values for overall and active microbial communities in amended and un-amended treatments 1-month post-application, as determined using SSU rRNA analysis from RNA and DNA extracts.

	Azoxystrobin concentration			
	0 mg kg^−1^ DNA	25 mg kg^−1^ DNA	0 mg kg^−1^ RNA	25 mg kg^−1^ RNA
Fungi	2.047^a^	1.633^b^	1.855^ab^	1.152^c^
Bacteria	2.767^a^	2.691^a^	2.621^a^	2.660^a^
Nematodes	1.936^ab^	2.061^a^	1.639^a^	1.771^ab^

LSD = 0.379 (*p* = <0.05). Each diversity value represents the mean of 5 experimental replicates. Significantly different values between treatments are shown by different letters.

## References

[b0005] Adetutu E.M., Ball A.S., Osborn A.M. (2008). Azoxystrobin and soil interactions: degradation and impact on soil bacterial and fungal communities. J. Appl. Microbiol..

[b0010] Allison S.D., Martiny J.B.H. (2008). Resistance, resilience, and redundancy in microbial communities. PNAS.

[b0015] Amato M., Ladd J.N. (1988). Assay for microbial biomass based on ninhydrin-reactive nitrogen in extracts of fumigated soils. Soil Biol. Biochem..

[b0025] Bartlett D.W., Clough J.M., Godwin J.R., Hall A.A., Hamer M., Parr-Dobrzanski B. (2002). The strobilurin fungicides. Pest Manage. Sci*.*.

[b0030] Bending G.D., Rodríguez-Cruz M.S., Lincoln S.D. (2007). Fungicide impacts on microbial communities in soils with contrasting management histories. Chemosphere.

[b0035] Bonkowski M. (2004). Protozoa and plant growth: the microbial loop in soil revisited. New Phytol..

[b0040] Boucard T.K., Parry J., Jones K., Semple K.T. (2004). Effects of organophosphate and synthetic pyrethroid sheep dip formulations on protozoan survival and bacterial survival and growth. FEMS Microbiol. Ecol..

[b0045] Degens B.P., Schipper L.A., Sparling G.P., Duncan L.C. (2001). Is the microbial community in a soil with reduced catabolic diversity less resistant to stress or disturbance?. Soil Biol. Biochem..

[b0050] Doick K.J., Lee P.H., Semple K.T. (2003). Assessment of spiking procedures for the introduction of a phenanthrene-LNAPL mixture into field-wet soil. Environ. Pollut..

[b0055] Edel-Hermann V., Gautheron N., Alabouvette C., Steinberg C. (2008). Fingerprinting methods to approach multitrophic interactions among microflora and microfauna communities in soil. Biol. Fert. Soils.

[b0060] Ekelund F. (1999). The impact of the fungicide fenpropimorph (Corbel®) on bactivorous and fungivorous protozoa in soil. J. Appl. Ecol..

[b0065] Ekelund F., Westergård K., Søe D. (2000). The toxicity of the fungicide propiconazole to soil flagellates. Biol. Fert. Soils.

[b0070] El Fantroussi S., Verschuere L., Verstraete W., Top E.M. (1999). Effect of phenylurea herbicides on soil microbial communities estimated by analysis of 16S rRNA gene fingerprints and community-level physiological profiles. Appl. Environ. Microbiol..

[b0075] Engelen B., Meinken K., von Wintzingerode F., Heuer H., Malkomes H.-P., Backhaus H. (1998). Monitoring impact of a pesticide treatment on bacterial soil communities by metabolic and genetic fingerprinting in addition to conventional testing procedures. Appl. Environ. Microbiol..

[b0080] Fierer N., Jackson J.A., Vigalys R., Jackson R.B. (2005). Assessment of soil microbial community structure by use of taxon-specific quantitative PCR assays. Appl. Environ. Microbiol..

[b0090] Gardes M., Bruns T.D. (1993). ITS primers with enhanced specificity for basidiomycetes – application to the identification of mycorrhizae and rusts. Mol. Ecol..

[b0095] Giller K.E., Witter E., McGrath S.P. (1998). Toxicity of heavy metals to microorganisms and microbial processes in agricultural soils: a review. Soil Biol. Biochem..

[b0105] Girvan M.S., Campbell C.D., Killham K., Prosser J.I., Glover L.A. (2005). Bacterial diversity promotes community stability and functional resilience after perturbation. Environ. Microbiol..

[b0110] Hackl E., Zechmeister-Boltenstern S., Bodrossy L., Sessitsch A. (2004). Comparison of diversities and compositions of bacterial populations inhabiting natural forest soils. Appl. Environ. Microbiol..

[b0125] Hunter P.J., Hand P., Pink D., Whipps J.M., Bending G.D. (2010). Both leaf properties and microbe-microbe interactions influence within-species variation in bacterial population diversity and structure in the lettuce (*Lactuca* species) phyllosphere. Appl. Environ. Microbiol..

[b0130] Janssen P.H., Yates P.S., Grinton B.E., Taylor P.M., Sait M. (2002). Improved culturability of soil bacteria and isolation in pure culture of novel members of the divisions Acidobacteria, Actinobacteria, Proteobacteria, and Verrucomicrobia. Appl. Environ. Microbiol..

[b0135] Joergensen R.G., Brookes P.C. (1990). Ninhydrin-reactive nitrogen measurements of microbial biomass in 0.5 M K_2_SO_4_ soil extracts. Soil Biol. Biochem..

[b0160] Mikola J., Bardgett R.D., Hedlund K., Loreau M., Naeem S., Inchausti P. (2002). Biodiversity, ecosystem functioning and soil decomposer food webs. Biodiversity and Ecosystem Functioning: Synthesis and Perspectives.

[b0165] Northcott G.L., Jones K.C. (2000). Spiking hydrophobic compounds into soil and sediment: a review and critique of adopted procedures. Environ. Toxicol. Chem..

[b0170] OECD, 2011. OECD Guidelines for the Testing of Chemicals (Online). URL: <http://www.oecd.org/document/7/0,3343,en_2649_34377_37051368_1_1_1_1,00.html>.

[b0175] O’Shaughnessy J.B., Chan M., Clark K., Ivantich K.M. (2003). Primer design for automated DNA sequencing in a core facility. Biotechniques.

[b0180] Ramette A. (2007). Multivariate analyses in microbial ecology. FEMS Microbial. Ecol..

[b0195] Seybold C.A., Herrick J.E., Brejda J.J. (1999). Soil resilience: a fundamental component of soil quality. Soil Sci..

[b0205] Spedding T.A., Hamel C., Mehuys G.R., Madramootoo C.A. (2004). Soil microbial dynamics in maize-growing soil under different tillage and residue management systems. Soil Biol. Biochem..

[b0210] Stanley Alliance Info-Tech, 2011. The Story of the Strobilurin Fungicide. Online. AgroNews. URL: <http://www.news.agropages.com/WiKi/Detail---4388.htm>.

[b0215] Tabatabai, M.A., 1994. Soil enzymes. In: Weaver, R.W., Angel, G.S., Bottomley, P.S., (Eds.), Methods of Soil Analysis: Chemical and Microbiological Properties, Part 2. Soil Science Society of America Book Series No. 5, Madison, USA, pp. 775–833.

[b0220] Toyota K., Ritz K., Young I.M. (1996). Survival of bacterial and fungal populations following chloroform-fumigation: effects of soil matric potential and bulk density. Soil Biol. Biochem..

[b0225] Urich T., Lanzén A., Qi J., Huson D.H., Schleper C., Schuster S.C. (2008). Simultaneous assessment of soil microbial community structure and function through analysis of the meta-transcriptome. PLoS ONE.

[b0230] Wang M.-C., Liu Y.-H., Wang Q., Gong M., Hua X.-M., Pang Y.-J., Hu S., Yang Y.-H. (2008). Impacts of methamidophos on the biochemical, catabolic, and genetic characteristics of soil microbial communities. Soil Biol. Biochem..

[b0235] Whelan J.A., Russell N.B., Whelan M.A. (2003). A method for the absolute quantification of cDNA using real-time PCR. J. Immunol. Methods.

[b0240] Zhang B., Bai Z., Hoefel D., Tang L., Wang X., Li B., Li Z., Zhuang G. (2009). The impacts of cypermethrin pesticide application on the non-target microbial community of the pepper plant phyllosphere. Sci. Total Environ..

[b0245] Zhang Y., Zhang X., Zhang H., He Q., Zhou Q., Su Z., Zhang C. (2009). Responses of soil bacteria to long-term and short-term cadmium stress as revealed by microbial community analysis. B. Environ. Contam. Toxicol..

[b0250] Zhang B., Deng H., Wang H.-L., Yin R., Hallett P.D., Griffiths B.S., Daniell T.J. (2010). Does microbial habitat or community structure drive the functional stability of microbes to stresses following re-vegetation of a severely degraded soil?. Soil Biol. Biochem..

