# Supplementary material for: Resistance and resilience responses of a range of soil eukaryote and bacterial taxa to fungicide application
Source: Chemosphere. 2014 Oct;112:194–202. doi: 10.1016/j.chemosphere.2014.03.031 (PMC4286127; doi:10.1016/j.chemosphere.2014.03.031)
Supplement: Supplementary data 1 [file mmc1.docx]

| **Organism**  **Group** | **Forward Primer** | **Reverse Primer** | **Annealing Temperature** | **Approximate Fragment Size (bp)** | **Restriction Enzyme(s) for T-RFLP** |
| --- | --- | --- | --- | --- | --- |
| **Fungi** | **EF4f-FAM:** 5' GGA AGG G(G/A)T GTA TTT ATT AG 3  (Smit *et al*., 1999) | **EF3r:** 5' GTT TGA ACC AGT AAA TCT CCT 3'  (Smit *et al*., 1999) | 48°C | 1,400 | *Hha*I, *Msp*I |
| **Bacteria** | **63f-NED:** 5' CAG GCC TAA CAC ATG CAA GTC 3'  (Marchesi *et al.*, 1998) | **1087r-VIC:** 5' CTC GTT GCG GGA CTT ACC CC 3’  (Hauben *et al*., 1997) | 55°C | 1,000 | *Hha*I, *Msp*I |
| **Archaea** | **AR3f:** 5' TTC CGG TTG ATC CTG CCG GA 3'  (Giovannoni, 1988) | **AR927r-NED:** 5' CCC GCC AAT TCC TTT AAG TTT C 3'  (Jurgens *et al*., 1997) | 55°C | 600 | *Hha*I, *Alu*I |
| **Pseudomonads** | **PS16Sf-FAM:** 5' ACT GAC ACT GAG GTG CGA AAG GC 3'  (Locatelli *et al*., 2002) | **PS23Sr:** 5' ACC GTA TGC GCT TCT TCA CTT GAC C 3'  (Locatelli *et al*., 2002) | 55°C | 750 | *Hha*I, *Alu*I |
| **Nematodes** | **Nem18Sf-VIC:** 5' CGC GAA T(G/A) G CTC ATT ACA ACA GC 3'  (Floyd *et al*., 2005) | **Nem18Sr:** 5' GGG CGG TAT CTG ATC GCC 3'  (Floyd *et al.*, 2005) | 56°C | 900 | *Aci*I, *Hae*III |

Supplementary Information

Table S1. The primer pairs used for community structure analysis

**References**

Floyd, R.M. et al., 2005. Nematode-specific PCR primers for the 18S small subunit rRNA gene. Mol. Ecol. Notes 5: 611-612.

Giovannoni, S.J. et al., 1988. Phylogenetic group-specific oligodeoxynucleotide probes for identification of single microbial-cells. J. Bacteriol. 170: 720-726.

Hauben, L. et al., 1997. Comparison of 16S ribosomal DNA sequences of all *Xanthomonas* species. Int. J. Syst. Evol. Micr. 47(2): 328-335.

Jurgens, G. et al., 1997. Novel group within the kingdom *Crenarchaeota* from boreal forest soil. Appl. Environ. Microbiol. 63(2): 803-805.

Locatelli, L. et al., 2002. Specific PCR amplification for the genus *Pseudomonas* targeting the 3’ half of 16S rDNA and the whole 16S-23S rDNA spacer. Syst. Appl. Microbiol. 25(2): 220-227.

Marchesi, J.R. et al., 1998. Design and evaluation of useful bacterium-specific PCR primers that amplify genes coding for bacterial 16S rRNA. Appl. Environ. Microbiol. 64(2): 795-799.

Smit, E. et al., 1999. Analysis of fungal diversity in the wheat rhizosphere by sequencing of cloned PCR-amplified genes encoding 18S rRNA and temperature gradient gel electrophoresis. Appl. Environ. Microbiol. 65(6): 2614-2621.


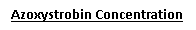

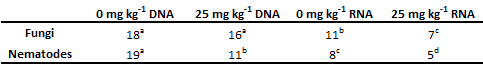


LSD = 2.459. Each number of TRFs represents the mean of 5 experimental replicates. Different letters denote significant differences between the treatments.

Table S2. Average TRF numbers for overall and active fungal and nematode communities in amended and un-amended treatments 1-month post application.

Figure S1

factor [levels=2] contrast

calc contrast=newlevels(conc;!(1,2,2,2,2))

"General Analysis of Variance."

BLOCK Rep

TREATMENTS Sample*(contrast/conc)

COVARIATE "No Covariate"

ANOVA [PRINT=aovtable,information,means; FACT=32; CONTRASTS=7; PCONTRASTS=7; FPROB=yes;\

PSE=diff,lsd; LSDLEVEL=5] diversity

APLOT [RMETHOD=simple] fitted,normal,halfnormal,histogram

AGRAPH [METHOD=means; PSE=differences] X=

Figure S1. An example of the Genstat code used to produce a modified ANOVA with an added contrast analysis function. The contrast function enabled enable the impacts of individual azoxystrobin concentrations on microbial communities to be compared with each other.
